# Supplementary material for: Explosive mutation accumulation triggered by heterozygous human Pol ε proofreading-deficiency is driven by suppression of mismatch repair
Source: eLife. 2018 Feb 28;7:e32692. doi: 10.7554/eLife.32692 (PMC5829921; doi:10.7554/eLife.32692)
Supplement: Figure 1—source data 1. — HCT-116 cells (37.4 × 106) were transduced with Pol ε rAAV and grown in the presence of 10 μg/ml G418 to select for Neor clones. Targeted clones were identified by PCR analysis. [file elife-32692-fig1-data1.pptx]

## Slide 1
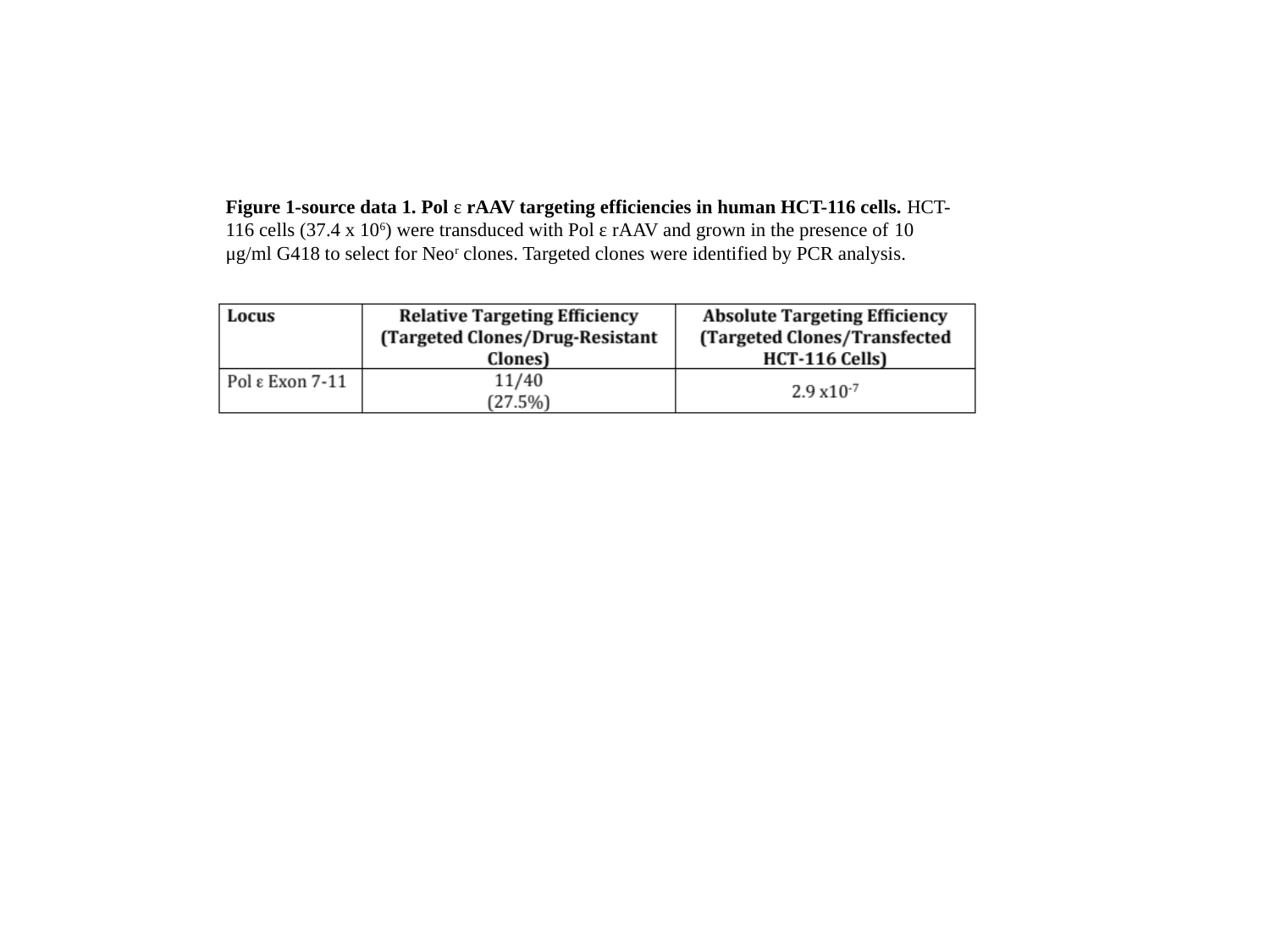

Figure 1-source data 1. Pol ε rAAV targeting efficiencies in human HCT-116 cells. HCT-116 cells (37.4 x 106) were transduced with Pol ε rAAV and grown in the presence of 10 μg/ml G418 to select for Neor clones. Targeted clones were identified by PCR analysis.
