# Supplementary material for: Explosive mutation accumulation triggered by heterozygous human Pol ε proofreading-deficiency is driven by suppression of mismatch repair
Source: eLife. 2018 Feb 28;7:e32692. doi: 10.7554/eLife.32692 (PMC5829921; doi:10.7554/eLife.32692)
Supplement: Figure 1—source data 2. — For each cell line, HPRT1 cDNA was made by RT-PCR, amplified and sequenced from independent 6-thioguanine resistant clones. Verified errors are indicated by type on the coding strand and position relative to the +1 start site. Insertion (ins) or deletion (Δ) of the indicated base(s) is denoted. [file elife-32692-fig1-data2.pptx]

## Slide 1
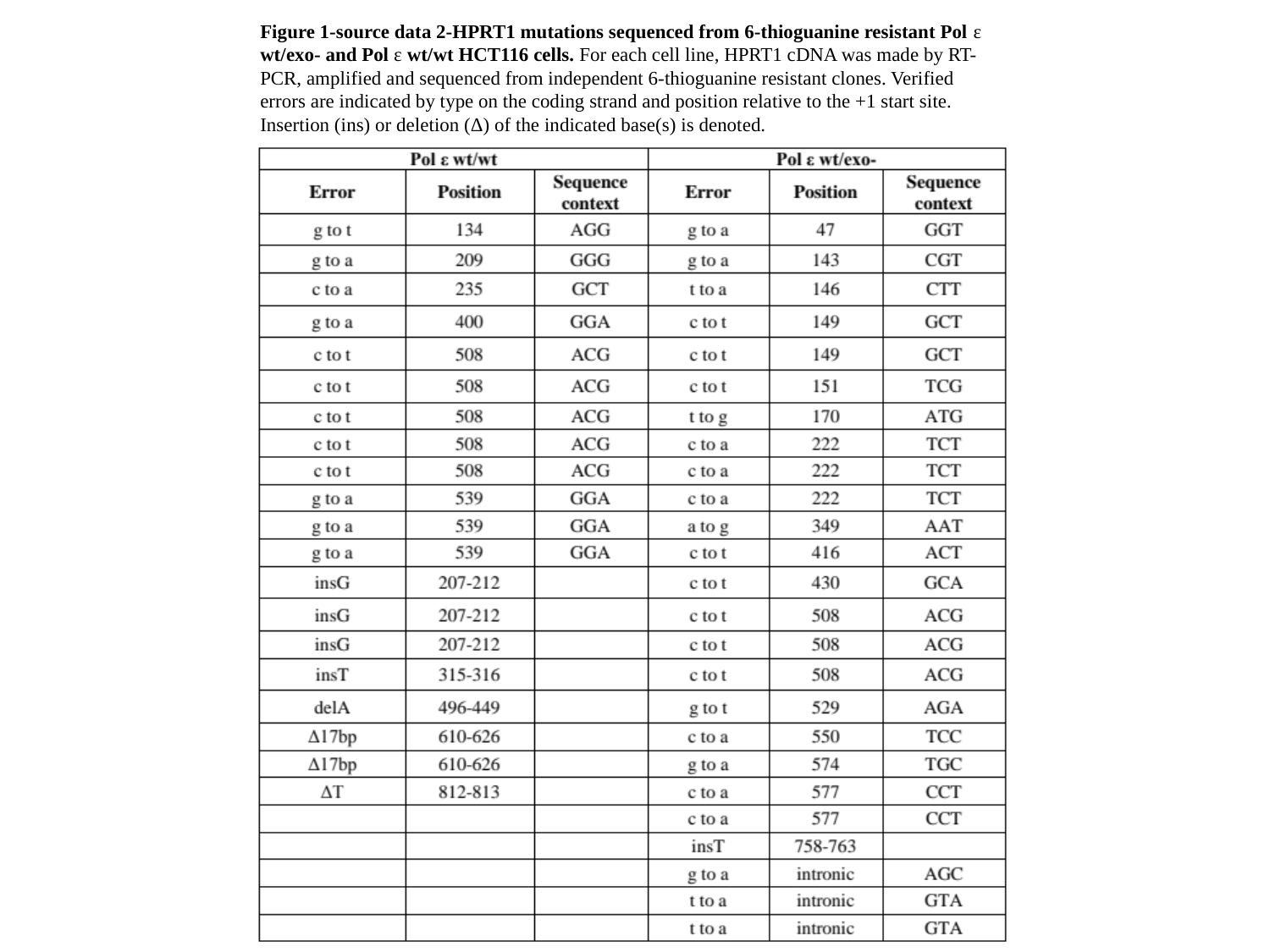

Figure 1-source data 2-HPRT1 mutations sequenced from 6-thioguanine resistant Pol ε wt/exo- and Pol ε wt/wt HCT116 cells. For each cell line, HPRT1 cDNA was made by RT-PCR, amplified and sequenced from independent 6-thioguanine resistant clones. Verified errors are indicated by type on the coding strand and position relative to the +1 start site. Insertion (ins) or deletion (Δ) of the indicated base(s) is denoted.
