# Supplementary material for: Explosive mutation accumulation triggered by heterozygous human Pol ε proofreading-deficiency is driven by suppression of mismatch repair
Source: eLife. 2018 Feb 28;7:e32692. doi: 10.7554/eLife.32692 (PMC5829921; doi:10.7554/eLife.32692)
Supplement: Figure 4—source data 1. — The HPRT1 ORF was sequenced from individual HPRT1-resistant clones at the indicated PDL as described (Figure 4A and Materials and methods). One clone was unable to be sequenced (n.d.). [file elife-32692-fig4-data1.pptx]

## Slide 1
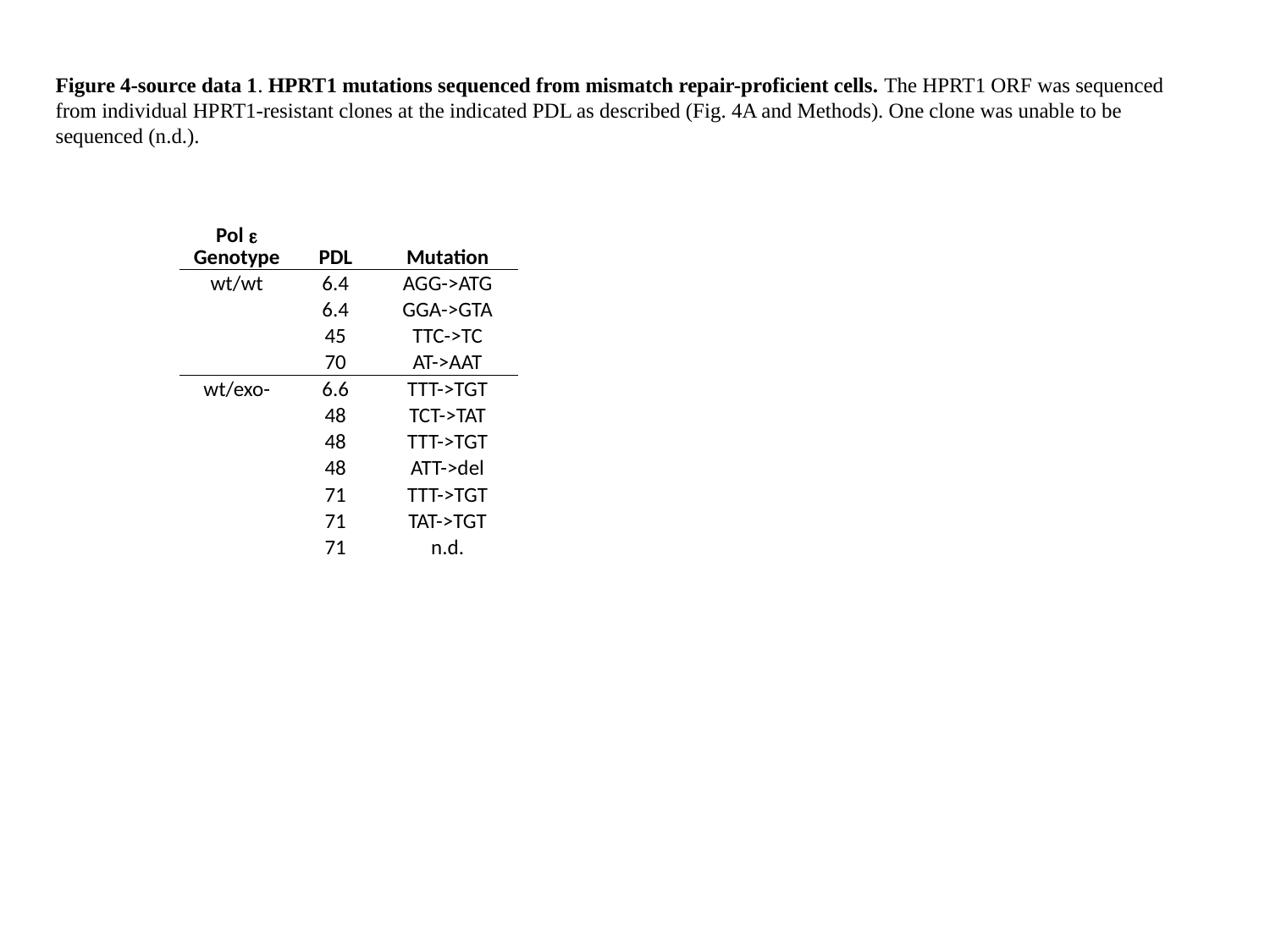

Figure 4-source data 1. HPRT1 mutations sequenced from mismatch repair-proficient cells. The HPRT1 ORF was sequenced from individual HPRT1-resistant clones at the indicated PDL as described (Fig. 4A and Methods). One clone was unable to be sequenced (n.d.).
| Pol e Genotype | PDL | Mutation |
| --- | --- | --- |
| wt/wt | 6.4 | AGG->ATG |
| | 6.4 | GGA->GTA |
| | 45 | TTC->TC |
| | 70 | AT->AAT |
| wt/exo- | 6.6 | TTT->TGT |
| | 48 | TCT->TAT |
| | 48 | TTT->TGT |
| | 48 | ATT->del |
| | 71 | TTT->TGT |
| | 71 | TAT->TGT |
| | 71 | n.d. |
