# Supplementary material for: Explosive mutation accumulation triggered by heterozygous human Pol ε proofreading-deficiency is driven by suppression of mismatch repair
Source: eLife. 2018 Feb 28;7:e32692. doi: 10.7554/eLife.32692 (PMC5829921; doi:10.7554/eLife.32692)
Supplement: Figure 4—source data 2. — Cosine similarities were calculated between the six unique mutation signatures extracted from POLE tumors and Pol ε mutant cell lines (columns, from Figure 2—figure supplement 2A) and each of the 30 identified Cosmic mutation signatures (http://cancer.sanger.ac.uk/cancergenome/assets/signatures_probabilities.txt). [file elife-32692-fig4-data2.pptx]

## Slide 1
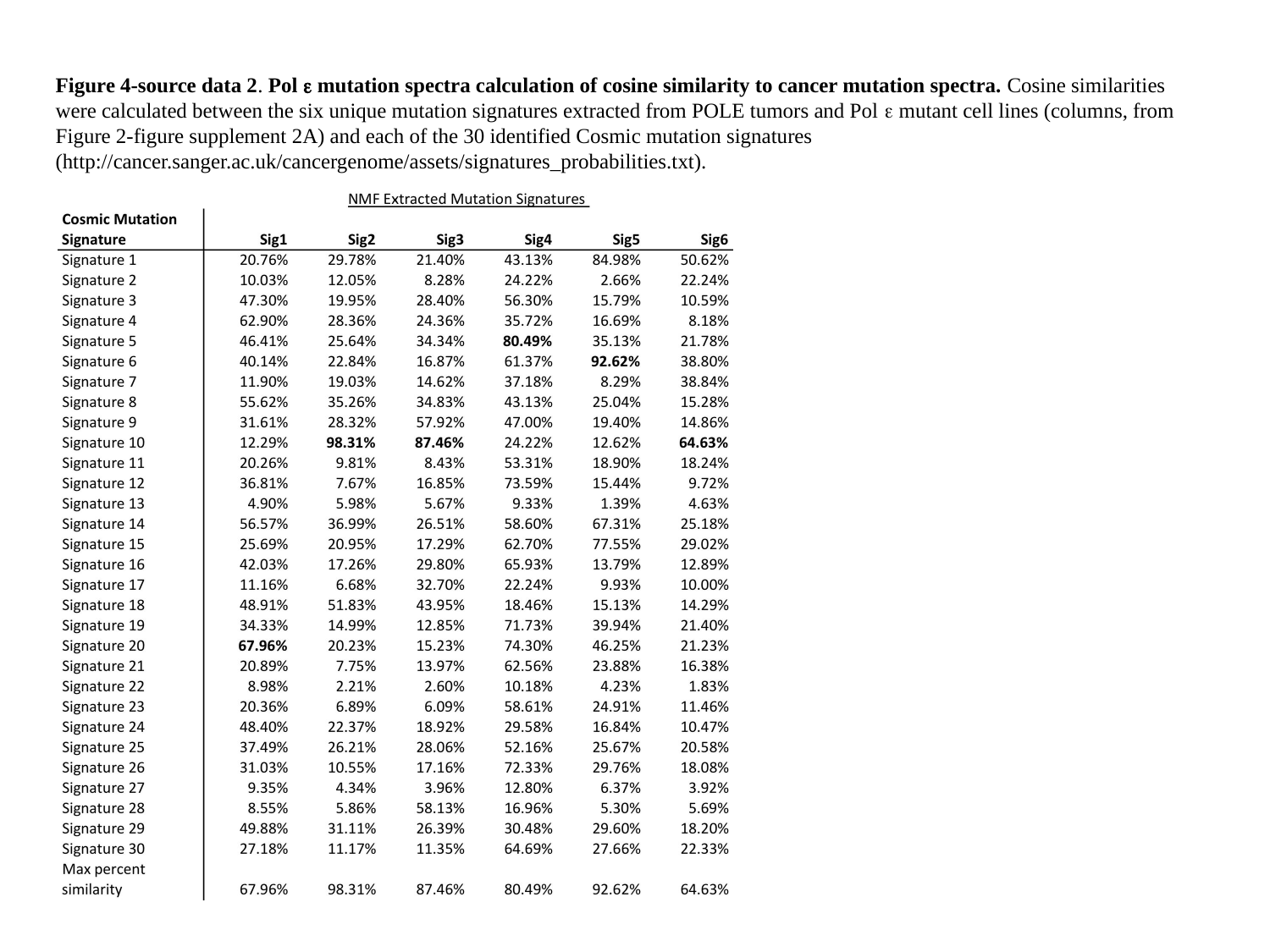

Figure 4-source data 2. Pol e mutation spectra calculation of cosine similarity to cancer mutation spectra. Cosine similarities were calculated between the six unique mutation signatures extracted from POLE tumors and Pol e mutant cell lines (columns, from Figure 2-figure supplement 2A) and each of the 30 identified Cosmic mutation signatures (http://cancer.sanger.ac.uk/cancergenome/assets/signatures_probabilities.txt).
