# Supplementary material for: Explosive mutation accumulation triggered by heterozygous human Pol ε proofreading-deficiency is driven by suppression of mismatch repair
Source: eLife. 2018 Feb 28;7:e32692. doi: 10.7554/eLife.32692 (PMC5829921; doi:10.7554/eLife.32692)
Supplement: Transparent reporting form [file elife-32692-transrepform.pdf]

eLIFE
1st Floor
24 Hills Road
Cambridge CB2 1JP, UK
P 01223 855340
W elifesciences.org
T @elife

eLife's transparent reporting form

We encourage authors to provide detailed information *within their submission* to facilitate the interpretation and replication of experiments. Authors can upload supporting documentation to indicate the use of appropriate reporting guidelines for health-related research (see EQUATOR Network), life science research (see the BioSharing Information Resource), or the ARRIVE guidelines for reporting work involving animal research. Where applicable, authors should refer to any relevant reporting standards documents in this form.

If you have any questions, please consult our Journal Policies and/or contact us: editorial@elifesciences.org.

Sample-size estimation

- You should state whether an appropriate sample size was computed when the study was being designed
- You should state the statistical method of sample size computation and any required assumptions
- If no explicit power analysis was used, you should describe how you decided what sample (replicate) size (number) to use

Please outline where this information can be found within the submission (e.g., sections or figure legends), or explain why this information doesn't apply to your submission:

No explicit power analysis was used when the study was being designed. However, care was taken to control for potential phenotypic variability by the decision to derive independent biological isolates for the mutation rate studies in Figure 1 and 3. Additionally, assay design required the replicate of three (3) for plating efficiency and twelve (12) for 6TG-selection performed in three separate experiments to determine mutation rates.

Replicates

- You should report how often each experiment was performed
- You should include a definition of biological versus technical replication
- The data obtained should be provided and sufficient information should be provided to indicate the number of independent biological and/or technical replicates
- If you encountered any outliers, you should describe how these were handled
- Criteria for exclusion/inclusion of data should be clearly stated
- High-throughput sequence data should be uploaded before submission, with a private link for reviewers provided (these are available from both GEO and ArrayExpress)

Please outline where this information can be found within the submission (e.g., sections or figure legends), or explain why this information doesn't apply to your submission:

eLife Sciences Publications, Ltd is a limited liability non-profit non-stock corporation incorporated in the State of Delaware, USA, with company number 5030732, and is registered in the UK with company number FC030576 and branch number BR015634 at the address 1st Floor, 24 Hills Road, Cambridge CB2 1JP | August 2014

Figure 1A-Mutation rates for each biological replicate were performed with 12 independent parallel cultures as is standard for fluctuation analyses. 6TG and plating efficiency technical replicates are described in Materials and Methods. Figure 1D-TCT>TAT reversion mutation rate biological and technical replicates are described in Figure 1 Legend. Figure 2A-For the NGS sequencing analyses, no biological or technical replicates were used. However, the data presented for POLE PD14 represent sequencing the same cell population once at PD0 and again 14 population doublings later. The results shown are the mutations found only in PD14. Figure 3B- Mutation rates for each biological replicate were performed with 12 independent parallel cultures as is standard for fluctuation analyses. 6TG and plating efficiency technical replicates are described in Materials and Methods. Figure 4A-PDL-specific mutant frequency technical replicates described in Materials and Methods and Figure 4 Legend. Figure 4B-For the NGS sequencing analyses, no biological or technical replicates were used. However, the data presented for POLE PD69 and PD71 represent sequencing the same cell population once at PD0 and again 69 and 71 population doublings later for the indicated cell populations. The results shown are the mutations found only in PD69 or PD71. Biological replicate refers to a cell line of the same genotype generated from independent drug-resistance cassette integration events (See Supp. Fig. 1B), Technical replicate refers to the repeat of the experiment with the same sample.
WES and WGS data is available through NCBI GEO under Accession: PRJNA327240 (Link: https://www.ncbi.nlm.nih.gov/bioproject/PRJNA327240/)

Statistical reporting

- Statistical analysis methods should be described and justified
- Raw data should be presented in figures whenever informative to do so (typically when N per group is less than 10)
- For each experiment, you should identify the statistical tests used, exact values of N, definitions of center, methods of multiple test correction, and dispersion and precision measures (e.g., mean, median, SD, SEM, confidence intervals; and, for the major substantive results, a measure of effect size (e.g., Pearson's r, Cohen's d)
- Report exact p-values wherever possible alongside the summary statistics and 95% confidence intervals. These should be reported for all key questions and not only when the p-value is less than 0.05.

Please outline where this information can be found within the submission (e.g., sections or figure legends), or explain why this information doesn't apply to your submission:

Figure 1B-Unpaired student's t-test was performed, additional statistical data present in Figure 1 Legend. Figure 1D-Statistical data present in Figure 1 Legend. Figure 2A-Statistical data present in Figure 2 Legend, n.s. p-value = 0.8551; *** designation for HCC2998 C>A refers to $X^2 = 872$ df = 1; *** designation for POLE wt/exo- PD14 C>A refers to $X^2 = 2368$ df = 1. Figure 3B-Unpaired student's t-test, additional statistical data present in Figure 1 and 3 Legend. Figure 4A-Unpaired student's t-test was performed n.s. p-value = 0.3439. Figure 4B-Statistical data present in Figure 4 Legend, *** designation for POLE wt/exo- C>A refers to p-value = 0.0002.

(For large datasets, or papers with a very large number of statistical tests, you may upload a single table file with tests, Ns, etc., with reference to sections in the manuscript.)

Group allocation

- Indicate how samples were allocated into experimental groups (in the case of clinical studies, please specify allocation to treatment method); if randomization was used, please also state if restricted randomization was applied
- Indicate if masking was used during group allocation, data collection and/or data analysis

Please outline where this information can be found within the submission (e.g., sections or figure legends), or explain why this information doesn't apply to your submission:

Our experiments did not warrant any group allocation as treatment was consistent across all samples.

Additional data files ("source data")

- We encourage you to upload relevant additional data files, such as numerical data that are represented as a graph in a figure, or as a summary table
- Where provided, these should be in the most useful format, and they can be uploaded as "Source data" files linked to a main figure or table
- Include model definition files including the full list of parameters used
- Include code used for data analysis (e.g., R, MatLab)
- Avoid stating that data files are "available upon request"

Please indicate the figures or tables for which source data files have been provided:

Source data available for: Figure 1A, 1B and 1C-In Supp. Table 2 and Supp. Table 3. Figure 2A and 2B-At the above NCBI GEO Accession number: PRJNA327240. Figure 4A-In Supp. Table 5. Figure 4B-At the above NCBI GEO Accession number: PRJNA327240
